# Epidemiological landscape and genetic prospects for marker-assisted selection in Kazakh sheep

**DOI:** 10.3389/fvets.2025.1647959

**Published:** 2025-08-26

**Authors:** Shynggys Orkara, Aigerim Khamzina, Nurlan Sandybayev, Rano Sattarova, Ainur Davletova, Kadyrzhan Khamzin, Kisun Pokharel, Primkul Ibragimov, Nadezhda Burambayeva, Darkhan Smagulov

**Affiliations:** ^1^Kazakh National Agrarian Research University, Almaty, Kazakhstan; ^2^Laboratory of Bacteriology, LLP “Kazakh Scientific Research Veterinary Institute”, Almaty, Kazakhstan; ^3^West Kazakhstan Innovation and Technological University, Uralsk, Kazakhstan; ^4^LLP Scientific and Educational Center “Qazyna”, Almaty, Kazakhstan; ^5^Natural Resources Institute Finland (LUKE), Jokioinen, Finland; ^6^Faculty of Agricultural Sciences, NJSC “Toraigyrov University”, Pavlodar, Kazakhstan

**Keywords:** Kazakh sheep, diseases, genetic resistance, genetic markers, associative studies

## Abstract

This comprehensive review examines the current epidemiological landscape, genetic resistance mechanisms, and control strategies for major sheep diseases in Kazakhstan. The study systematically analyzes three primary disease categories: parasitic infestations (including echinococcosis, fascioliasis, gastrointestinal strongylatosis, and protozoan infections), infectious diseases (foot rot, scrapie, Maedi-Visna, pasteurellosis, brucellosis), and hereditary disorders. Our analysis revealed significant regional variations in disease prevalence, with southern and western regions of Kazakhstan exhibiting higher parasitic burdens, particularly for echinococcosis and fascioliasis. Recent molecular studies have identified promising genetic markers associated with disease resistance, notably polymorphisms in the MHC complex (particularly *DRB1* and *DQB1* loci) conferring protection against parasitic infections and the *PRNP* gene variants influencing scrapie susceptibility. Current disease control approaches in Kazakhstan predominantly rely on chemical treatments and vaccination programs, while genetic selection for disease resistance remains underutilized despite its demonstrated efficacy in international breeding programs. The integration of marker-assisted selection and genomic approaches represents a promising strategy for enhancing disease resistance while maintaining productivity in Kazakhstani sheep breeds. This review highlights critical knowledge gaps, particularly regarding the molecular epidemiology of hereditary disorders as a markers of ecological plasticity and the genetic basis of resistance to infectious diseases in local breeds, emphasizing the need for comprehensive surveillance programs and targeted research to develop sustainable disease management strategies for Kazakhstan’s sheep industry.

## 1 Introduction

Sheep farming is one of the key sectors of animal husbandry in Kazakhstan, making a significant contribution to the national economy through the production of meat, wool, and milk. Kazakhstan holds a leading position in Central Asia in terms of sheep population and ranks 16th globally among lamb producers, accounting for 1.2% of global production (155.4 thousand tons per year) (1). According to the Bureau of National Statistics of the Republic of Kazakhstan, the total sheep population in the country reached 23,1 million in 2025 year. Historically and culturally ingrained in Kazakhstan’s rural communities, sheep farming faces considerable challenges from various disease agents that threaten animal welfare, productivity, and ultimately, food security. Despite advances in veterinary medicine, sheep diseases continue to cause substantial economic losses. The geographical diversity of Kazakhstan spanning arid steppes, semi-deserts, and mountainous regions—creates varied ecological niches for pathogen persistence and transmission. These environmental conditions, combined with traditional extensive grazing practices, facilitate the maintenance of complex disease cycles involving multiple hosts and vectors (2–4). Furthermore, Kazakhstan’s position at the crossroads of Eurasia presents unique epidemiological challenges, particularly regarding transboundary diseases and emerging pathogens.

Parasitic diseases remain among the most economically significant health issues affecting sheep in Kazakhstan. Echinococcosis, fascioliasis, dicrocoeliosis, and gastrointestinal strongylatosis display concerning prevalence rates across different regions, with notable hotspots in the southern and western territories (5). These parasitoses not only diminish production parameters but several, including echinococcosis, also pose significant zoonotic threats to public health (6).

Infectious diseases constitute another major challenge, with foot rot, scrapie, Maedi-Visna, pasteurellosis, brucellosis causing considerable morbidity and mortality. While Kazakhstan has implemented surveillance and control programs for notifiable diseases like brucellosis the epidemiological landscape remains dynamic, with periodic outbreaks highlighting gaps in preventive strategies (7). The recent increase in brucellosis seroprevalence from 4.2% in 2020 to 14.9% in 2021 illustrates the persistent challenge of controlling such infectious agents despite ongoing vaccination programs (8–10).

Hereditary diseases, though less extensively documented in Kazakhstani sheep populations, represent an emerging area of concern as intensive selection for production traits may inadvertently increase the frequency of deleterious alleles. Conditions such as scrapie susceptibility, spider lamb syndrome, entropion, and various metabolic disorders have been identified in sheep globally, with potential implications for Kazakhstani breeds (11–14).

The conventional approach to disease management in Kazakhstan has predominantly relied on chemical treatments for parasites and vaccination programs for infectious diseases. However, these strategies face increasing challenges, including anthelmintic resistance, vaccine failure, and environmental concerns. Under these circumstances, the use of genetic resistance is being considered as a promising and environmentally safe complement to existing disease control methods.

This review aims to synthesize current knowledge on the epidemiology, genetic basis of resistance, and control strategies for major sheep diseases in Kazakhstan. By critically analyzing parasitic, infectious, and hereditary conditions, was identified knowledge gaps and integrated approaches to disease management that combine conventional veterinary interventions with genetic selection. Such a holistic strategy is essential for developing resilient sheep populations capable of withstanding diverse disease challenges while meeting the growing demand for sustainable animal products in Kazakhstan and beyond.

## 2 General classification of sheep diseases and resistance

Parasitic and infectious diseases represent one of the most serious challenges in sheep farming, significantly affecting both the economic stability of livestock enterprises and public health. These diseases not only reduce animal productivity but also complicate the production of high-quality and safe products, including meat and dairy, which are crucial for ensuring human health and well-being.

Parasitic diseases such as oestrosis, echinococcosis, monieziasis, coenurosis, dictyocaulosis, coccidiosis, wohlfahrtiosis, dicrocoeliasis, and fascioliasis cause significant damage to the industry (Table 1). They lead to weight loss, stunted growth and development, and, in severe cases, death, exacerbating the economic difficulties faced by livestock farmers (15).

**Table 1:** Parasitic diseases of sheep: pathogens, symptoms, and regional distribution in Kazakhstan.

Disease	Pathogen/Parasites	Symptoms	Region
Oestrosis	*Oestrus ovis* (fly larvae)	Nasal and respiratory discharge	Akmola
Echinococcosis	*Echinococcosis granulosus*	Liver/lung cysts, weight loss	West Kazakhstan
Monieziasis	*Moniezia expansa* (tapeworn)	Diarrhea, growth retardation	Akmola
Coenurosis	*Taenia multiceps* larva	Neurological sings	Rare
Dictyocaulosis	*Dictyocaulus filaria*	Coughing, dyspnea	Almaty, Akmola
Coccidiosis	*Eimeria* spp.	Diarrhea, dehydration	Zhambyl
Dicrocoeliasis	*Dicrocoelium dendricticum*	Liver disfunction	Zhambyl
Fascioliasis	*Fasciola hepatica/gigantica*	Liver damage, anemia	West Kazakhstan, Almaty

At the same time, infectious diseases such as scrapie, lentiviral infections, foot rot, pasteurellosis, brucellosis also contribute significantly to the decline in sheep health and productivity. Acute outbreaks of these diseases can result in mass mortality, reduced product quality, and substantial costs associated with treatment and prevention (15). Table 2 summarizes the infectious diseases affecting sheep, their pathogens, symptoms, and available resistance information.

**Table 2:** Sheep infectious diseases: pathogens, symptoms, and resistance information.

Disease	Pathogen/Parasites	Type	Symptoms
Scrapie	*Prion*	Neurodegenerative	Ataxia, behavioral changes
Lentiviral Infections	*Visna-Maedi virus (small ruminant lentivirus)*	Viral	Pneumonia, mastitis, encephalitis
Foot Rot	*Dichelobacter nodosus* and *Fusobacterium necrophorum*.	Bacterial	Lameness, Hoof damage
Pasteurellosis	*Pasteurella multocida, Mannheimia haemolytica*	Bacterial	Septicemia, respiratoy issues
Brucellosis	*Brucella melitensis*	Bacterial	Abortion, infertility

Research aimed at identifying genetic markers associated with disease resistance often employs the candidate gene approach (16–20). This method involves analyzing the association between variations in specific genes involved in the host’s immune response and resistance to infections. The most frequently studied markers have been identified in the major histocompatibility complex (MHC) region on ovine chromosome 20 (*Ovis aries*, OAR20). Genes in this region exhibit high polymorphism and play a key role in antigen presentation to the host’s T lymphocytes, triggering a cascade of immune responses (21–23).

Quantitative trait locus (QTL) mapping and genome-wide association studies (GWAS) are also used to study disease resistance. Both approaches enable the identification of genomic regions influencing economically significant traits such as disease resistance. QTL studies are conducted on populations with known pedigrees and use a limited number of genetic markers, such as microsatellites (STRs). In contrast, GWAS utilizes thousands of single nucleotide polymorphisms (SNPs), allowing precise identification of genomic regions associated with target traits, even in the absence of pedigree data (21). SNP microarrays allow animals to be genotyped at multiple loci across the genome (up to 50,000 loci), enabling detailed analysis of genetic profiles even in the absence of pedigree information. These approaches are particularly useful for analyzing data collected on farms during disease outbreaks. For example, infected and control groups can be genotyped, and based on the obtained data, genetic profiles of the animals can be constructed (21).

### 2.1 Parasitic diseases

Parasitic diseases significantly impact livestock health and productivity, causing economic losses and posing zoonotic risks (24). These conditions are caused by protozoa, helminths (nematodes, cestodes, trematodes), and ectoparasites (ticks, mites, lice), which affect various organs and systems. In ruminants, parasitic infections often lead to poor growth, reduced feed efficiency, reproductive issues, and even death (25).

Genetic markers determining sheep resistance to gastrointestinal parasites have long been a focus in livestock breeding, as their use allows for the selection of more resistant individuals and a reduction in parasite transmission within the flock. Polymorphisms associated with the major histocompatibility complex (MHC) and interferon (IFN)-*γ* genes are most frequently cited in studies as key markers influencing susceptibility to infection (21, 26, 27). Other studies have identified cytokines such as IL4, IL5, IL13RA2, and IL13, which play a crucial role in immune response to parasitic infections like *Teladorsagia circumcincta* (28). At the molecular level, resistance to parasites is associated with differential expression of immune response genes. Transcriptomic analysis of abomasal tissue from sheep of two lines selectively bred for resistance to *Haemonchus contortus* (HSF and TSF) revealed between 11 and 903 differentially expressed genes, depending on genetic background and immune response type (innate or adaptive). Particularly significant were genes involved in the Th2 immune response, such as *PRKG1, CALM2, MYL1*, and *ITGB1*, as well as those in signaling pathways activating both innate and adaptive immunity (17).

GWAS in Corriedale sheep using various SNP marker panels (170 K, 507 K, and 50 K SNPs) identified several quantitative trait loci (QTL) associated with resistance to gastrointestinal nematodes. The most significant QTL were located on chromosomes 1, 3, 12, and 19. Of particular interest are genomic regions on chromosomes 1 and 3 harboring *TIMP3*, *TLR5*, *LEPR*, and *TLR9* genes, which are potentially involved in immune response regulation and account for a substantial proportion of the observed variation in fecal egg counts among the studied animals (29).

#### 2.1.1 Echinococcosis

Echinococcosis, caused by the larval stage of the cestode *Echinococcus granulosus*, remains one of the most common parasitic infestations of sheep in Kazakhstan. According to results of comprehensive epizootiological monitoring conducted in three regions of the republic in 2013 (Almaty - 23%, Zhambyl - 25%, South Kazakhstan - 35%, and West Kazakhstan - 28.4%), the infection rate of sheep with echinococcosis ranged from 23.0 to 35.0% (25).

**Figure 1:**
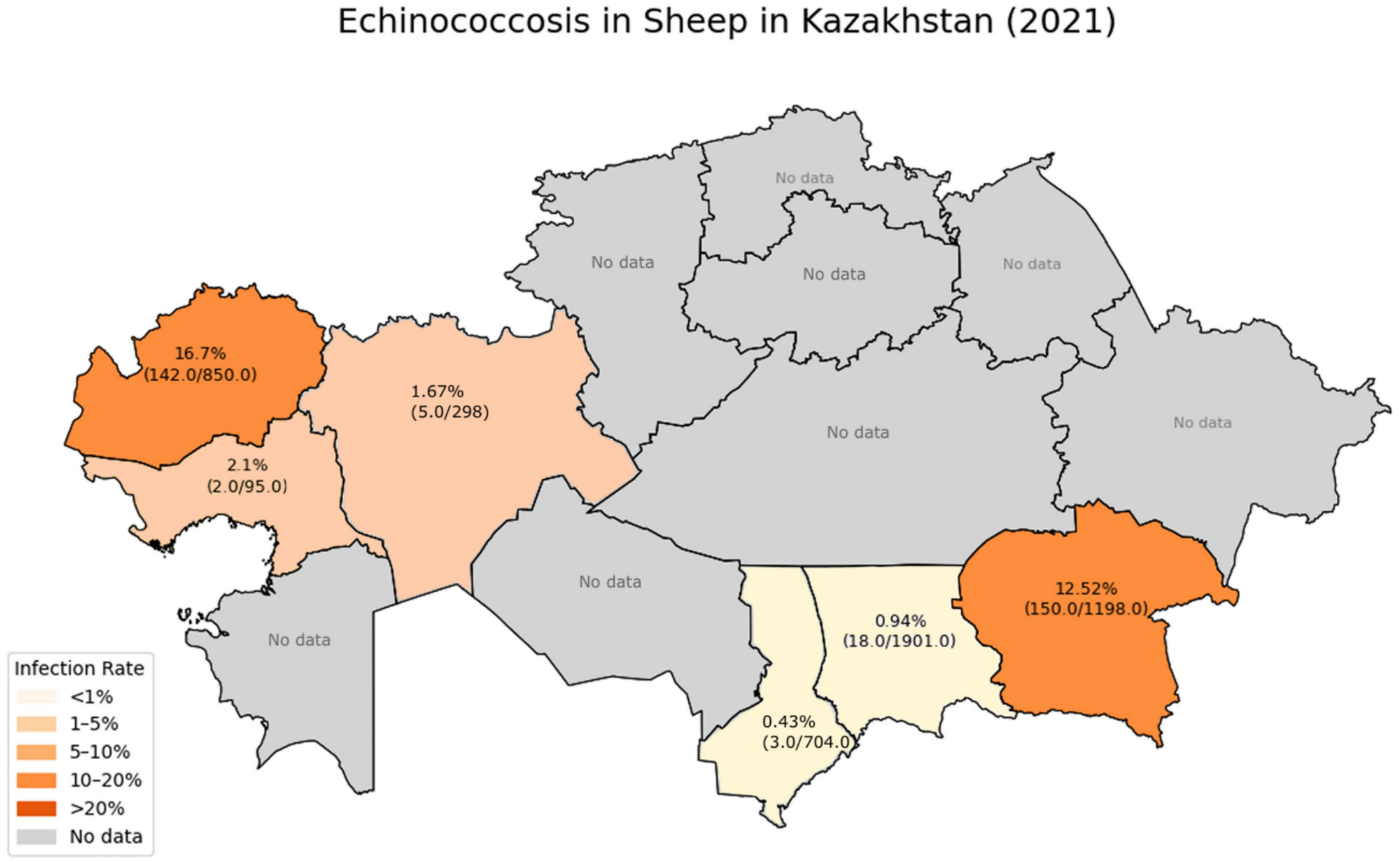
Prevalence of echinococcosis in sheep by Region.

According to a study conducted in 2021 (6), the prevalence of echinococcosis in sheep by region was as follows in Figure 1.

These data indicate a significant reduction in the incidence of sheep infections in the Zhambyl, Turkestan, Aktobe, and Atyrau regions compared to previously published figures, which showed that the infection rate in southern regions reached 30–50% in past years. Specifically, in 2003, the prevalence of echinococcosis in sheep in the Almaty, Zhambyl, and South Kazakhstan regions ranged from 30 to 50% (30). According to data published in 2010, the infection rate in South Kazakhstan fluctuated between 24 and 32%. In Almaty region, the 2010 data showed an infection rate ranging from 7.7 to 69% (6).

However, in the Almaty and West Kazakhstan regions, the infection rates remain high, indicating active circulation of the pathogen in the “sheep-dog” transmission cycle and insufficient effectiveness of the control measures being implemented.

According to the research findings, in the overwhelming majority of infected sheep (84.66%), the cysts were localized in the liver, with a less frequent occurrence in the lungs (10%), and in 5.33% of cases, a mixed localization (liver and lungs) was observed. The intensity of the infestation ranged from 1 to 12 cysts per animal (6).

Despite the persistently high infection rates in regions genetic factors influencing host resistance remain underexplored in local sheep populations. A study by Hong Shen et al. (20) on Chinese Merino sheep established a link between polymorphisms in *the MHC-DRB1* and *MHC-DQB1* genes and resistance or susceptibility to cystic echinococcosis. Certain genotypes of these genes were associated with resistance (MvaIbb, HaeIIIee, SacIab, TaqIaa, HaeIIInn, MvaIdz) or susceptibility (SacIIab, HaeIIIdf, HaeIIIbd, MvaIbc, TaqIab, HaeIIImn, MvaIcz) to the disease. Special attention was given to the DRB1-SacIab/DRB1-MvaIbb/DQB1-TaqIaa/DQB1-HaeIIInn haplotype, which demonstrated a strong association with resistance to cystic echinococcosis. This haplotype was experimentally confirmed: in a group of sheep carrying the specified haplotype, the infection rate was significantly lower compared to the control group (20).

Deeper investigations into the genetic basis of resistance to parasitic infections have revealed that MHC class II genes govern the recognition of parasitic antigens and the initiation of adaptive immune responses. Studies by Li et al. (17) demonstrated that polymorphisms in exon 2 of the *DRB1* gene in Kazakh sheep exhibit a statistically significant correlation (*p* < 0.01) with resistance to echinococcosis. The most influential genotypes were MvaIbc, Hin1Iab, and SacIIab, with carriers showing markedly lower infection rates compared to sheep possessing the MvaIbb, SacIIaa, Hin1Ibb, HaeIIIef (*p* < 0.01), and HaeIIIab (*p* < 0.05) genotypes. Experimental challenge trials confirmed that the *MvaIbc-SacIIab-Hin1Iab* haplotype is associated with resistance (*p* < 0.01) (17).

#### 2.1.2 Fascioliasis and dicrocoeliasis

Fascioliasis, caused by the trematodes *Fasciola hepatica* and *F. gigantica*, is widely distributed among sheep in all the studied regions of Kazakhstan. According to veterinary services, fascioliasis is diagnosed in 9–11% of animals during post-slaughter inspections in Kazakhstan (5). At the same time, most sources report that the infection rate among productive animals can range from 18 to 50%. According to research conducted by Abdrakhmanov et al. (5) in the Terekty district of the West Kazakhstan region, the coprological detection rate in a study of 374 sheep samples showed an infestation rate of 40.8%, or 158 positive samples. Earlier studies also recorded high infection rates in other regions (31). For example, in the Almaty region, the infection rate was 23.8% with an infestation intensity of up to 3 eggs, in the Abay district of the East Kazakhstan region 4.8% with an intensity of up to 3 eggs, and in the Turkestan region, the infection rate in sheep with *F. gigantica* was 3.6%, while with *F. hepatica* it was 2.8%. In a comparative aspect, the level of sheep infection with fascioliasis in Kazakhstan is significantly lower than in South Asian and North African countries, where the infection rate reaches 53.5% in Pakistan, 26.4% in China, and 22.2% in Egypt (17, 32–35).

Studies conducted on Indonesian thin-tail sheep demonstrated breed-specific resistance to *F. gigantica*, correlating with the dominance of a Th1-mediated immune response. Resistant animals showed significantly increased expression of IL-12p40 and IL-2 in hepatic lymph nodes at early stages of infection, as well as a lower IL-4/IFN-*γ* ratio compared to susceptible sheep. Resistant Indonesian thin-tail sheep exhibited a predominance of IgG2 antibodies, whereas susceptible Merino sheep were characterized by a pronounced Th2 response with dominance of IL-4, IL-5, and IL-13, along with an increased IgG1/IgG2 ratio (*p* < 0.05) (36).

Dicrocoeliasis, caused by *Dicrocoelium dendriticum*, is also recorded in all the studied regions, with an infestation rate ranging from 3.2 to 7.0% (25). According to other sources, the infestation rate of sheep across the country reaches 9% (37). The highest infection rates with dicrocoeliosis have been observed in the Zhambyl region.

#### 2.1.3 Gastrointestinal strongylatosis

Gastrointestinal strongylatosis refers to a group of helminthiases caused by nematodes of the suborder *Strongylata*, and is characterized by the highest prevalence among sheep helminthiases in Kazakhstan. According to coprological studies, the infestation rate ranges from 28.8% in the Zhambyl region to 53.0% in the Almaty region (25). Recent studies in the Akmolinsk region revealed an infection rate of *Trichostrongylidae* spp. at 77.1%, with a high intensity of infestation (1,100 ± 98 eggs/g) (38). Strongylatosis is often recorded as part of mixed infestations, exacerbating the pathogenic effect on the host’s body and complicating diagnosis and therapy.

The high prevalence of strongylatosis is due to a combination of factors, including favorable climatic conditions for the development of infective larvae, the predominance of pasture-based sheep farming, and the insufficient effectiveness of preventive deworming. The economic damage caused by this group of helminthiases results from reduced meat productivity, poorer wool quality, and increased susceptibility of infected animals to infectious diseases (39).

#### 2.1.4 Cestodoses

Monieziosis, caused by tapeworms of the *Moniezia* and *Thysaniezia genera*, is recorded in all the studied regions, with an infestation rate ranging from 4.4 to 10.9% Specifically, in the Akmolinsk region, *Moniezia* spp. were found in 23% of animals (average intensity – 350 ± 38 eggs/g) (38). This infestation primarily affects young sheep, causing growth and development delays, and in severe cases, animal death.

#### 2.1.5 Blood parasitic diseases

Blood parasitic infections in sheep, caused by *Babesia ovis* and *Theileria ovis*, present a serious problem for sheep farming in Kazakhstan, especially in the southern and southeastern regions of the country, where favorable conditions for the development of tick vectors have formed (40). According to official veterinary reports for the Turkestan region from 2018 to 2023, 34,630 cases of piroplasmosis among small ruminants were registered, with Theileriosis (41.9% of total cases) being more prevalent than Babesiosis and Piroplasmosis (29.3%) (41).

Studies conducted in Southern India established an association between certain alleles of the *Ovar-DRB1* gene and sheep resistance to Theileriosis. It was found that the ‘b’ allele, both in the heterozygous (ab) and homozygous (bb) states, was significantly more common in clinically healthy animals, suggesting its potential role in the genetic resistance to the pathogen (42).

#### 2.1.6 Eimeriosis

Eimeriosis, caused by protozoa of the *Eimeria genus*, also represents a significant problem for sheep farming, particularly in intensive livestock farming conditions. In the Zhambyl region, the infestation rate of sheep with Eimeria reaches 45.8%, indicating the need for improvements in anti-parasitic measures for this infestation (25).

The results of epidemiological monitoring show the widespread occurrence of parasitic infestations in sheep across all the studied regions of Kazakhstan, with the highest infestation rates of gastrointestinal strongylatosis (28.8–53.0%) and echinococcosis (23.0–35.0%) (25, 38). Identified molecular genetic markers of resistance to echinococcosis (haplotype MvaIbc-SacIIab-Hin1Iab of the *Ovar-DRB1* gene) and fasciolosis (association with Th1-mediated immune response) are of significant interest for the development of breeding programs aimed at increasing sheep resistance to parasitic diseases (20, 36). A comprehensive approach, including regular epidemiological monitoring, molecular genetic research, and the improvement of anti-parasitic measures, will significantly reduce the economic damage caused by parasitic diseases to sheep farming in Kazakhstan.

#### 2.1.7 Prevention and treatment measures

Breeding sheep using genetic markers for parasite resistance is a promising direction for improving both productivity and overall animal health. Genetic resistance to parasitic infestations, especially gastrointestinal nematodes, allows for a significant reduction in the use of anthelmintic drugs and minimizes the risk of resistance development in parasites.

Studies show that resistance to helminth infections has moderate heritability, making effective selection possible (43, 44). Specifically, in the Blond-faced Manech sheep breed, the heritability of resistance to nematodes is 0.18, allowing for the use of genetic selection to enhance resistance (44). It is important to note that genetic correlations between resistance to parasites and productive traits vary widely, which opens up opportunities for comprehensive selection (45).

Genomic selection, based on the use of multiple molecular markers, shows even higher effectiveness. In a study conducted on Merino sheep in Australia, the inclusion of genomic information in breeding value evaluation models using the ssGBLUP (single-step genomic BLUP) method increased the average individual accuracy of selection predictions by 4% for animals with genotypes and phenotypes, and by 8% for individuals lacking phenotypic data but with available genomic information (46). These data highlight the potential of using genomic data to improve the effectiveness of breeding programs.

Vaccination against parasitic diseases in sheep also relies on understanding the genetic mechanisms of the immune response. The development of recombinant subunit vaccines against *Haemonchus contortus*, containing the antigens H11 and H-gal-GP, has demonstrated high efficacy (80–90%) under field conditions (47, 48).

For the prevention of echinococcosis, a vaccine based on the recombinant antigen EG95 is successfully used, showing an efficacy of up to 98% in vaccinated animals (49). Genetic studies have revealed that variations in genes of the major histocompatibility complex (MHC) class II, particularly in the DQB1 locus, correlate with resistance or susceptibility to echinococcosis. Specifically, in Chinese Merino sheep, the genotypes DQB1-TaqIaa and DQB1-HaeIIInn are associated with resistance to the disease, while the genotypes DQB1-TaqIab and DQB1-HaeIIImn are associated with susceptibility to infection (50).

An innovative approach in the prevention of sheep fascioliasis is the development of vaccines based on proteolytic enzymes from the parasite. Vaccination of sheep with cathepsins L (CL1 and CL2) and leucine aminopeptidase (LAP) isolated from adult forms of *Fasciola hepatica* demonstrated significant protective efficacy: the combined use of CL1 and CL2 provided 60% protection, while a triple combination of CL1, CL2, and LAP provided up to 78% protection. The highest level of protection (89%) was achieved with the use of LAP alone, which induced both humoral and cellular immune responses (51). These data emphasize the importance of considering the genetic profile of animals when planning vaccination programs. Individual differences in MHC alleles can influence the effectiveness of the immune response to a vaccine, making genetic analysis a valuable tool for optimizing preventive measures and breeding programs.

Modern control programs for parasitic diseases in sheep are based on comprehensive strategies that integrate genetic selection methods with preventive programs. The inclusion of resistance to helminths in breeding indices, as implemented in the U. S. National Sheep Improvement Program (NSIP), enables simultaneous progress in both productive and immunological traits. For example, adding the resistance to gastrointestinal nematodes (assessed by fecal egg count, FEC) to breeding indices has reduced parasitic burden by 5–8% per year while maintaining up to 98% genetic gain in lamb weaning weight (52).

Such balanced selection demonstrates the effectiveness of a long-term approach to sustainable animal husbandry, aimed at reducing reliance on anthelmintic drugs and minimizing the risks of parasite resistance formation.

### 2.2 Infectious diseases

#### 2.2.1 Foot rot

Foot rot in sheep is an infectious disease caused by the bacteria *Dichelobacter nodosus* and *Fusobacterium necrophorum*. This disease is prevalent in most sheep- and goat-rearing countries. It leads to lameness, reduced productivity, and significant economic losses. The costs associated with combating foot rot exceed millions of dollars in various countries. For example, in the United Kingdom, the estimated annual cost is £24.4 million (53). In Switzerland, this disease results in an annual economic loss of 33 million Swiss francs (54). In the United States and Australia, losses are estimated at $18.4 million (55).

It is important to note that scientific publications on the prevalence and epizootiology of ovine footrot in Kazakhstan are extremely limited, highlighting the need for localized research on this topic.

A genome-wide association study (GWAS) on sheep resistance to foot rot conducted by Mehmet et al. (56) identified several significant genes playing a key role in disease resistance. One of these genes is *GBP6* (guanylate-binding protein 6), which is involved in phagocyte activation and initiates immune responses against bacterial pathogens (56).

Another crucial gene is *TCHH*, which contributes to the mechanical strength of hooves and wool. Mutations in this gene have been found to increase susceptibility to hoof damage, thereby promoting the development of foot rot.

Additionally, the *SLC38A1* gene, located on chromosome 3, was identified as being associated with glutamine transport and oxidative stress regulation. It is hypothesized that this gene can limit the availability of essential nutrients to pathogens such as *Dichelobacter nodosus*, thereby reducing the likelihood of infection (56).

##### 2.2.1.1 Diagnostic methods and control strategies

Modern approaches to diagnosing infectious diseases in small ruminants are based on a combination of clinical, laboratory, and molecular-genetic methods. The effectiveness of controlling these diseases depends on timely and accurate diagnostics, which necessitates the continuous improvement of the methods used (57–59).

The diagnosis of foot rot is based on a comprehensive approach that includes clinical examination and microbiological methods. Using the Egerton and Roberts scale (1971), the severity of the disease is rated from 1 to 5 points, where 5 points correspond to the complete detachment of the horn layer of the hoof. Modern diagnostics rely on the isolation and identification of the pathogens *Dichelobacter nodosus* and *Fusobacterium necrophorum* using cultural methods and PCR diagnostics (60–62).

The multiplex PCR (32) allows for the simultaneous detection and differentiation of virulent and avirulent strains of *Dichelobacter nodosus* based on the presence of *aprV2/B2* genes, which encode thermostable proteases.

Best et al. (63) developed a loop-mediated isothermal amplification (LAMP) method for field diagnosis of *D. nodosus*, which provides results within 15–20 min, with a diagnostic specificity of 100% and sensitivity of 83.33% (detection threshold of 5×10^−3^ ng μL^−1^). LAMP tests show particular promise for rapid field diagnostics (63).

Foot rot control includes a range of measures: regular hoof trimming, treatment with hoof baths containing zinc sulfate (15–18%) or formalin (5%) (61). and isolation of infected animals. Antibiotics are used in acute cases, with high efficacy observed for oxytetracycline, lincomycin, and gamithromycin (64–66).

Additionally, strategies such as genetic selection for resistance to foot rot or phenotypic selection based on the condition of hooves in healthy and affected animals are applied (67, 68).

##### 2.2.1.2 Vaccination and epidemiological monitoring

The development of effective vaccines against footrot is complicated by the considerable antigenic variability of *D. nodosus*, which is classified into 10 serogroups (A-I and M) (69). Currently, two main vaccination strategies are employed: the use of multivalent vaccines, which include up to nine serogroups (A–I), and the application of bivalent vaccines targeted at the most common isolates (such as H and B).

Research findings presented by McPherson et al. (70) suggest that bivalent vaccines induce a higher and more sustained level of antibodies compared to multivalent vaccines. This advantage is attributed to the elimination of antigenic competition when a two-month interval between vaccinations is maintained. Field evaluations of vaccine effectiveness showed that the duration of immune protection provided by multivalent vaccines is shorter than that of bivalent vaccines, despite the broader serogroup coverage (70).

On the other hand, an analysis by Raadsma and Dhungyel (69) demonstrates that the serogroup structure of infections within flocks varies significantly. Up to six different serogroups were identified within a single flock, with the overall prevalence of serogroups H and B exceeding 60% individually, but only 27% of flocks exclusively carried these two serogroups. This indicates that a universal bivalent vaccine provides complete protection only for a limited number of flocks (69). Thus, to achieve reliable immune prophylaxis, the use of flock-specific vaccines is recommended.

Regarding foot rot, monitoring includes clinical diagnostics, laboratory detection of the pathogen, and serotyping. According to Zanolari (61), the prevalence of the disease varies by country, ranging from low (Iran, Sweden) to high (Australia, the United Kingdom). Differences in diagnostic approaches (surveys, clinical examinations, PCR) complicate direct comparisons of data. In Switzerland, for example, annual non-material losses related to the deterioration of sheep welfare were estimated at 33 million Swiss francs (approximately USD 37.5 million). It has been established that breed characteristics (e.g., Merinos) can increase susceptibility, but the contribution of genetic factors to resistance is only 15–25%, which is outweighed by the influence of management conditions and flock management (61).

#### 2.2.2 Scrapie

Scrapie is a highly dangerous chronic disease of sheep and goats with an extended incubation period of up to six years. Like other transmissible spongiform encephalopathies, including Creutzfeldt-Jakob disease in humans and bovine spongiform encephalopathy (BSE), scrapie requires close monitoring and control.

Scrapie remains a serious problem for sheep farming worldwide, being an endemic disease in several countries with developed sheep farming, including the United Kingdom, Greece, Romania, and Germany, where animal susceptibility is associated with polymorphisms in codons 136, 154, and 171 of the *PRNP* gene. At the same time, sheep populations in China and Pakistan exhibit high genetic resistance to the disease, as evidenced by the absence of registered cases (71).

In Kazakhstan, no official cases of scrapie have been recorded. However, the lack of systematic research on the genetic profile of local sheep breeds prevents the definitive exclusion of potential risks. According to informal surveys of farmers, neurological disorders resembling scrapie (itching, ataxia, hyperexcitability) are occasionally observed in sheep herds. However, due to the absence of laboratory diagnostics and the practice of immediate culling of suspicious animals, verifying the diagnosis is challenging.

Given the economic significance of sheep farming for Kazakhstan and the potential risks of cross-border spread of prion diseases, conducting large-scale screening of *PRNP* gene polymorphisms in local breeds, as well as implementing monitoring programs for small ruminants, is highly relevant. This will allow for an accurate assessment of the epidemiological situation and the development of preventive measures, including the selection of animals with resistant genotypes.

Scrapie control strategies rely on studying polymorphisms in the PrP gene. A direct correlation between PrP polymorphisms and disease susceptibility has been established. The polymorphisms associated with the risk of classical scrapie are located at codons 136 (alanine/valine), 154 (arginine/histidine), and 171 (glutamine/histidine/arginine) of the PrP gene. These combinations form five main haplotypes: ARR, ARH, ARQ, AHQ, and VRQ, where ARR is associated with minimal risk and VRQ with the highest susceptibility (71, 72). The study of PrP gene polymorphisms in native sheep breeds is conducted in most countries worldwide (11, 73–76).

In sheep, the *PRNP* gene is located on chromosome 13 (genomic coordinates: NC_030820.1: 46,453,052–46,473,740), and encodes the prion protein, a key factor involved in neurodegenerative processes. The specific codon variants form the basis for genetic resistance or susceptibility, making this locus a critical target in national scrapie eradication programs. Genomic databases such as Homologene, Ensembl, and NCBI Gene provide comprehensive annotations supporting its role in disease development.

Data on sheep genotypes provide valuable insights for breeding programs aimed at selecting scrapie-resistant animals (71). In European countries, selection programs for scrapie resistance based on PrP genotyping began as early as 2005. In the United Kingdom, the Ram Genotyping Scheme operated from 2001 to 2009 and included more than 12,000 flocks (covering approximately 80% of ram-producing flocks). This program significantly altered PrP haplotype frequencies, leading to a reduction in scrapie prevalence between 2002 and 2012 (72).

Amara et al. (77) described a case of classical scrapie in a six-year-old Barbarine sheep in Tunisia. The animal exhibited clinical signs such as weight loss, pruritus, skin lesions, wool loss, and ataxia. Histopathological and immunohistochemical analyses revealed characteristic spongiform changes in the brain and deposits of pathological prion protein. The diagnosis was confirmed via Western blotting, which identified the classical PrPres molecular pattern, distinguishing it from atypical scrapie and BSE. Genetic analysis showed the sheep carried the ARQ/ARQ genotype, which is known to confer high susceptibility to scrapie (77).

##### 2.2.2.1 Diagnostic methods and control strategies

The diagnosis of scrapie presents significant challenges due to the long incubation period and variability in clinical manifestations (78). Live animal diagnosis includes immunohistochemical examination of third eyelid biopsies and lymphoid tissue from the rectum (79); ELISA for detecting pathological prions in biological fluids; and RT-QuIC (real-time quaking-induced conversion) analysis, which can detect minimal amounts of prion protein (80).

Postmortem diagnosis remains the gold standard for scrapie detection. It involves histopathological examination of brain tissue, followed by immunohistochemical analysis and Western blotting to confirm the presence of PrP^Sc^ (81). According to Dennis et al. (79), the immunohistochemical method for scrapie diagnosis in sheep has a sensitivity ranging from 85.3 to 89.4%.

Molecular-genetic typing based on polymorphisms in codons 136, 154, and 171 of the *PRNP* gene is a mandatory component of monitoring programs (82). According to the National Scrapie Eradication Program (NSEP), sheep are categorized into five risk groups (R1-R5) depending on their genotype (72).

Control Measures for Scrapie:

- Genetic selection for resistance using PRNP genotyping.
- Culling of infected animals and proper disposal of carcasses.
- Quarantine measures for herds with diagnosed cases.
- Restrictions on the movement and trade of animals without confirmed resistance genotypes.

Control programs for scrapie in European countries have shown the effectiveness of the genetic approach. In the UK, between 2001 and 2006, the frequency of scrapie cases decreased. Comparisons of prevalence and slaughterhouse screenings showed rates of 0.06 and 0.23% in 2003, which dropped to 0.02 and 0.17% in 2006, respectively, while the proportion of sheep with the resistant ARR/ARR genotype increased from 28.8 to 47.6% (72).

##### 2.2.2.2 Vaccination and epidemiological monitoring

Vaccination against prion diseases represents a complex challenge due to the unique pathogenetic characteristics and the absence of a classical immune response. Nevertheless, research in this area continues. An experimental vaccine based on polyomavirus-like particles (VLPs) displaying various prion peptides showed promising results in preclinical trials. In a study conducted by Eiden et al. (83), C57BL/6 mice were immunized with nine variants of prion peptides presented on the capsid proteins VP1/VP2 of hamster polyomavirus. After immunization, the animals were intraperitoneally infected with the RML strain of scrapie. The most effective of the tested vaccines resulted in a statistically significant increase in average lifespan—up to 240 days compared to 202 days in the control group (83). These data suggest the potential of using the VLP platform to develop effective vaccines against transmissible spongiform encephalopathies.

An alternative approach to combating prion diseases is the development of immunotherapeutic agents aimed at inducing autoantibodies against the normal cellular form of the prion protein (PrP^C). In a study by Abdelaziz et al. (84), the ability to overcome immune tolerance in transgenic mice expressing deer PrP was demonstrated through immunization with multimeric recombinant PrP proteins. All tested immunogens (mono- and dimers of PrP from mouse and deer) induced a high level of autoantibodies specific to PrP^C. Post-vaccination sera effectively bound to surface PrP on cells, blocked prion conversion in the RT-QuIC test, and inhibited the spread of the infectious agent in infected cells (84). These findings support the potential of active immunization using multimeric forms of PrP as a strategy for preventing chronic wasting diseases.

In the European Union, a two-tiered surveillance system for scrapie is in place: active and passive monitoring. According to Acín (85), three subgroups are subject to annual testing: (1) fallen animals over 18 months old, (2) cull animals as part of TSE measures, and (3) clinically healthy animals slaughtered for meat. An important element of control is the exclusion of specific risk materials (brain, spleen, ileum, etc.) from the food chain. Additionally, genetic monitoring is employed: in sheep populations, monitoring focuses on the increase of the ARR allele frequency, while in goat populations, it targets the distribution of alleles K222, D146, and S146, which are associated with resistance to classical scrapie (11, 72, 81).

#### 2.2.3 Maedi-Visna

Disease caused by small ruminant lentiviruses (SRLV), is recognized by the World Organization for Animal Health (WOAH) as a notifiable terrestrial animal disease due to its significant economic burden and detrimental impact on animal welfare (86, 87). The etiological agent, also referred to as Maedi/Visna virus (MVV), belongs to the genus *Lentivirus* within the family Retroviridae and induces a chronic multisystemic inflammatory disease in sheep. The most prominent clinical manifestation is progressive interstitial pneumonia accompanied by dyspnea, although the virus can also affect the central nervous system, mammary glands, and joints (88, 89). Transmission primarily occurs horizontally through respiratory secretions and the ingestion of infected colostrum and milk, with vertical transmission via the placenta and infection through semen also reported (59, 90). MVV exhibits high tropism for macrophages and shares virological features with HIV-1, albeit without inducing immunodeficiency. The disease typically follows a prolonged subclinical phase, culminating in irreversible pathological changes and death. Histopathological hallmarks include lymphoid hyperplasia, interstitial pneumonia, and, in some cases, necrotizing nonsuppurative encephalitis and poliomyelitis. Infected sheep display differential expression of host immune genes, including the upregulation of *CCR5*, *TLR7*, and *TLR8* in pulmonary lesions, indicating an active antiviral response (88). Despite its global distribution and severe outcomes, control of MV remains challenging due to the absence of effective antiviral therapies and the complexity of vaccine development, which is hindered by the virus’s high genetic variability and the ongoing emergence of novel genotypes and subtypes. The disease has been reported in various countries, including Spain, where a successful control program reduced seroprevalence in some flocks (91), and Japan, where MVV was first isolated in 2011 (92). Factors influencing MVV transmission include flock size, housing conditions, and weaving age (91). In Kazakhstan, the disease is not officially registered at the national level, and government funding for diagnostic activities related to MVV is currently lacking. However, its potential presence in sheep populations cannot be ruled out, indicating the need for further investigation and surveillance.

Research has identified *TMEM154* as a significant genetic factor influencing susceptibility to MVV. The E35K polymorphism in this gene is associated with infection risk: alleles encoding glutamate (E35) at position 35 increase susceptibility, while the lysine (K35) allele reduces the likelihood of infection. Sheep homozygous for the K35 allele are significantly less likely to be infected compared to animals with EK or EE genotypes (93–97).

In addition to *TMEM154*, White et al. (98) identified an insertion/deletion variant in the *LOC105603932* gene through an association study (98). Testing over 1,000 sheep revealed that homozygous animals had the lowest viral loads compared to other genotypes (*p* < 0.0001) (98). Two genes *TMEM154* and *LOC105603932* have been functionally characterized in relation to MMV resistance. The *TMEM154* gene, located on chromosome 17 (*Ovis aries*, NC_056070.1: 5175905–5,228,028), encodes a transmembrane protein involved in viral entry. Its E35K polymorphism is a known marker for MVV susceptibility: animals carrying the E35 allele exhibit increased risk of infection, whereas the K35 variant confers resistance. In contrast, *LOC105603932*, found on chromosome 20 (*Ovis aries*, NC_056073.1: 29567119–29,536,399), encodes a putative zinc finger protein implicated in immune regulation. A deletion variant within this gene has been significantly associated with reduced viral load in homozygous carriers, highlighting its potential as a marker for genetic resilience against MVV. These loci provide important targets for marker-assisted selection strategies aimed at enhancing disease resistance in sheep populations [Homologene, Ensembl, NCBI Gene].

A study conducted on 400 sheep from four native Sicilian breeds (Valle del Belìce, Comisana, Barbaresca, and Pinzirita) identified differences in *TMEM154* allele frequencies and MVV seroprevalence (98). The Barbaresca and Pinzirita breeds exhibited the lowest prevalence of MVV antibodies, which correlated with a high frequency of the protective K35 allele. In contrast, in the Valle del Belìce breed, which is selected primarily for milk production, the risk-associated E35 allele was more common. Data analysis demonstrated that sheep with EK and EE genotypes were more than three times as likely to be infected compared to those with the KK genotype (98).

##### 2.2.3.1 Diagnostic methods and control strategies

According to a meta-analysis of 58 studies conducted between 1981 and 2020, serological diagnosis has been the primary method for detecting Medi-Visna. Enzyme-linked immunosorbent assay (ELISA) is the most common screening method, with sensitivity ranging from 85 to 98% and specificity from 90 to 99%, depending on the viral strain and the antigenic composition of the test systems. Immunodiffusion in agar gel, though still in use, is less common due to its low sensitivity (99).

Reverse transcription PCR (RT-PCR) is also used to detect proviral DNA, though it has been reported in only 25% of studies. Combined diagnostic methods have proven to be highly effective. The combination of ELISA and PCR increases detection by 15–20% compared to using each test separately (99).

For disease prevention, genetic selection of animals can be applied. The selection of sheep for genetic resistance using marker information in the *TMEM154* genes represents a promising direction in disease prevention. A number of studies highlight the effectiveness of using such markers for selecting animals with resistance (18, 59).

##### 2.2.3.2 Vaccination and epidemiological monitoring

The development of effective vaccines against lentiviruses in small ruminants is complicated by significant genetic variability of the virus and the phenomenon of antibody-dependent enhancement of infection. Despite numerous attempts to create attenuated, subunit, and whole-virus vaccines, none of them have demonstrated sufficient effectiveness in protecting against the infection (87).

Current research is focused on utilizing the approach of reverse vaccinology. In a study by Koçkaya (100), an immunoinformatics evaluation of the gag (group-specific antigen) and env (envelope) proteins of MVV was conducted with the aim of constructing a multi-epitope vaccine. The gag protein demonstrated higher conservancy and antigenicity than the env protein and did not contain transmembrane domains. From the many predicted epitopes, only 19 were selected based on criteria such as antigenicity, non-allergenicity, non-toxicity, and solubility. The final multi-epitope vaccine construct showed high affinity for TLR-2/4, suggesting its potential ability to induce an innate immune response (100).

There are also studies on the development of DNA vaccines based on the introduction of plasmids encoding MVV antigens. According to Henriques et al. (101), such vaccines are capable of inducing both humoral and cellular immune responses.

Epidemiological monitoring of sheep diseases is a comprehensive set of measures aimed at assessing the prevalence, dynamics, and risk factors of infectious pathologies. In the case of maedi-visna, a key component of the monitoring is regular serological and molecular testing. According to Kalogianni (87), to achieve MVV-free status, 2 to 5 consecutive negative test results must be obtained over a period of 6, 12, or 24 months, depending on the country. Farms are classified by seroprevalence levels: high — over 70%, medium — 40–69%, low — 10–39%, very low — 1–9%, free — less than 1%. Control includes annual or more frequent sampling of breeding stock, removal of seropositive animals, artificial rearing of newborns, and spatial isolation of animals according to seroconversion status. In areas with high seroprevalence, the most effective practice is the annual culling of the oldest and lowest-producing seropositive animals, replacing them with seronegative individuals (87).

#### 2.2.4 Pasteurellosis

Pasteurellosis is an acute contagious disease primarily caused by *Pasteurella multocida* and *Mannheimia haemolytica*, affecting a wide range of domestic animals, including sheep and goats (58, 102). In small ruminants, the disease typically manifests in a respiratory form, often precipitated by stress factors such as transportation, overcrowding, sudden temperature fluctuations, or concurrent infections (103, 104). Clinical signs include hyperthermia (up to 41–42°C), dyspnea, serous to purulent nasal discharge, coughing, and general weakness (104). In peracute forms, animals may die suddenly without prior clinical manifestations. In lambs and kids, the infection can progress to septicemia, often resulting in high mortality within 24–48 h (104).

The economic impact of pasteurellosis on small ruminant farming in Kazakhstan is substantial, primarily due to animal losses, decreased productivity, and expenditures on therapeutic and preventive measures. Although the disease is relatively infrequent compared to its incidence in cattle, sporadic cases and localized outbreaks among sheep and goats are periodically recorded. For example, according to the Veterinary Department of the Almaty Region, a confirmed case of pasteurellosis in small ruminants was reported in early 2025 in the Enbekshikazakh district.

**Figure 2:**
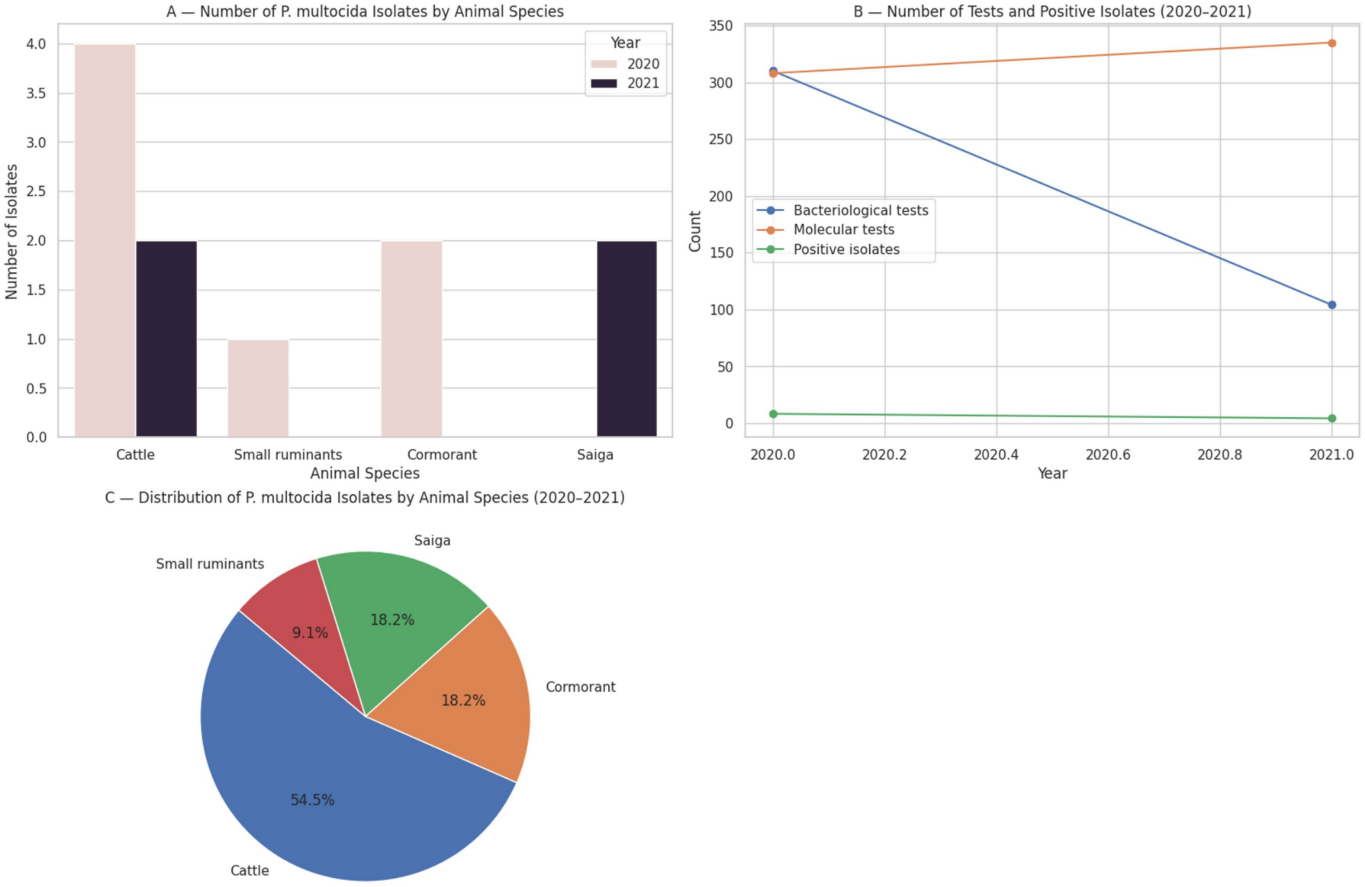
Surveillance data on *Pasteurella multocida* isolates by animal species and year, based on bacteriological and molecular testing conducted by the National Reference Center for Veterinary Medicine (Kazakhstan, 2020–2021).

Surveillance data collected by the National Reference Center for Veterinary Medicine (NRCVM, Astana, Kazakhstan) over two consecutive years illustrate the sporadic yet persistent detection of *Pasteurella multocida* in various animal species across Kazakhstan Figure 2 (8, 9).

In 2020, a total of 310 bacteriological and 308 molecular-biological investigations were conducted, resulting in the isolation of *P. multocida* in 8 cases. Among these, four isolates were obtained from cattle in the Burabay district of Akmola region, one from small ruminants in the Ili district of Almaty region, and two from great cormorants (*Phalacrocorax carbo*) collected along the Caspian coast in Atyrau region.

In comparison, the number of confirmed *P. multocida* isolates declined in 2021, with 104 bacteriological and 335 molecular-biological tests performed by the NRCVM, yielding four positive cases. These included two cases in cattle—one from Akmola region and one from Almaty region (Kegen district)—and two isolates from saiga antelope (*Saiga tatarica*) in the West Kazakhstan region (8).

These findings underscore both the host diversity and geographical distribution of pasteurellosis in Kazakhstan, with confirmed cases identified not only in domestic livestock (cattle and small ruminants) but also in wild species, such as saiga and cormorants. Notably, while cattle remain the primary species from which *P. multocida* is isolated, confirmed detections in small ruminants and wildlife indicate a broader epidemiological scope, reinforcing the need for multispecies surveillance under a One Health framework.

To date, there is a lack of published research specifically addressing the genetic resistance of sheep to pasteurellosis. Most existing studies have instead focused on the antimicrobial resistance profiles of the causative agents, particularly *Mannheimia haemolytica* and *Pasteurella multocida*, isolated from respiratory infections in sheep. These investigations have revealed varying levels of resistance to commonly used antibiotics, including high resistance to penicillin and oxytetracycline, as well as the presence of resistance-associated genes such as *strA* and *sul2*. While these findings underscore the importance of ongoing surveillance of antimicrobial resistance in pasteurellosis pathogens, they also highlight a significant research gap regarding the potential for inherited resistance to this disease in small ruminants (105, 106).

##### 2.2.4.1 Diagnostic methods and control strategies

*Pasteurella multocida* and *Mannheimia haemolytica* are key pathogens causing pneumonic pasteurellosis in sheep, characterized by clinical signs such as coughing, fever, and dyspnea (103). Diagnostic methods for this condition have evolved from traditional clinical and post-mortem examinations to more advanced molecular techniques. PCR-based methods, including real-time PCR, have emerged as highly sensitive and specific tools for detecting and identifying the causative agents (103). Additionally, acute phase proteins (haptoglobin, serum amyloid A) and proinflammatory cytokines (IL-1α, IL-1β, IL-6) have shown promising results as diagnostic biomarkers, demonstrating high accuracy in distinguishing infected from healthy sheep (103). These modern diagnostic approaches complement conventional methods like bacterial culture and biochemical tests, enhancing the accuracy and speed of diagnosis for pneumonic pasteurellosis in sheep.

Preventing and controlling pneumonic pasteurellosis in small ruminants requires a comprehensive approach that includes veterinary care, good hygiene, and proper management. Since Pasteurella and Mannheimia are opportunistic bacteria naturally present in the upper respiratory tract, complete prevention is difficult. Treatment is further complicated by mixed infections and growing antibiotic resistance, making sensitivity testing essential. Effective antibiotics include gentamicin, ceftriaxone, and long-acting oxytetracycline. Key control measures involve reducing stress, early diagnosis, targeted treatment, vaccination of healthy animals, and strict biosecurity. However, vaccine development is hindered by high serotype variability and lack of cross-protection, so control strategies must be adapted to local conditions (102, 107).

##### 2.2.4.2 Vaccination and epidemiological monitoring

Recent studies have investigated the efficacy of ovine pasteurellosis vaccines. A field trial in England found fewer mortalities in lambs vaccinated with a multivalent clostridial and Pasteurella vaccine compared to unvaccinated controls (107). However, a preliminary study in Ethiopia showed no significant difference in clinical signs or pathological findings between vaccinated and unvaccinated lambs challenged with *Mannheimia haemolytica* A1 (108). In Iraq, evaluation of a *Pasteurella multocida* biotype A vaccine revealed limited protective antibody titers against prevalent serotypes. Cross-protection studies demonstrated that a *Mannheimia haemolytica* serotype 1 vaccine provided considerable protection against a serotype 2 challenge in lambs, with vaccinated animals showing reduced pyrexia, lower dyspnoea scores, and smaller lung lesions compared to controls (109). These findings highlight the complexity of ovine pasteurellosis vaccination and the need for further research to improve vaccine efficacy.

#### 2.2.5 Brucellosis

Brucellosis is a globally prevalent zoonotic disease with significant implications for both animal health and public health, particularly within sheep-rearing regions. The disease is caused by *Brucella ovis* and *Brucella melitensis*, a bacterium that primarily affects livestock (110). A recent meta-analysis reported an overall seroprevalence of 6.2% in sheep across America, Africa, and Asia, with the highest regional rate observed in Africa (8.5%) (111). Prevalence varies substantially by location, with studies documenting seropositivity rates of 52% in Bogor, Indonesia (112), 15% in Bikaner, India (113), and 4.5% in El-Gadarif, Sudan (114). Clinically, brucellosis in sheep is often manifested by reproductive disorders, including late-term abortions, stillbirths, retained placenta, epididymitis in rams, and reduced fertility, which contribute significantly to economic losses in affected flocks. Identified risk factors associated with increased seroprevalence include adult age, history of abortion, flock size exceeding 20 animals, extensive (free-range) production systems, and mixed grazing with other species (111). In addition to its direct economic impact on livestock productivity, brucellosis presents a considerable public health burden due to its zoonotic nature.

In the Republic of Kazakhstan, brucellosis in farm animals remains a critical veterinary concern, with ongoing epizootic activity observed across nearly all administrative regions (115). Laboratory surveillance continues to be a cornerstone of the national brucellosis control strategy. A comparative analysis of laboratory data from 2020 to 2021 demonstrated both an expansion in diagnostic capacity and persistent challenges in disease detection.

**Figure 3:**
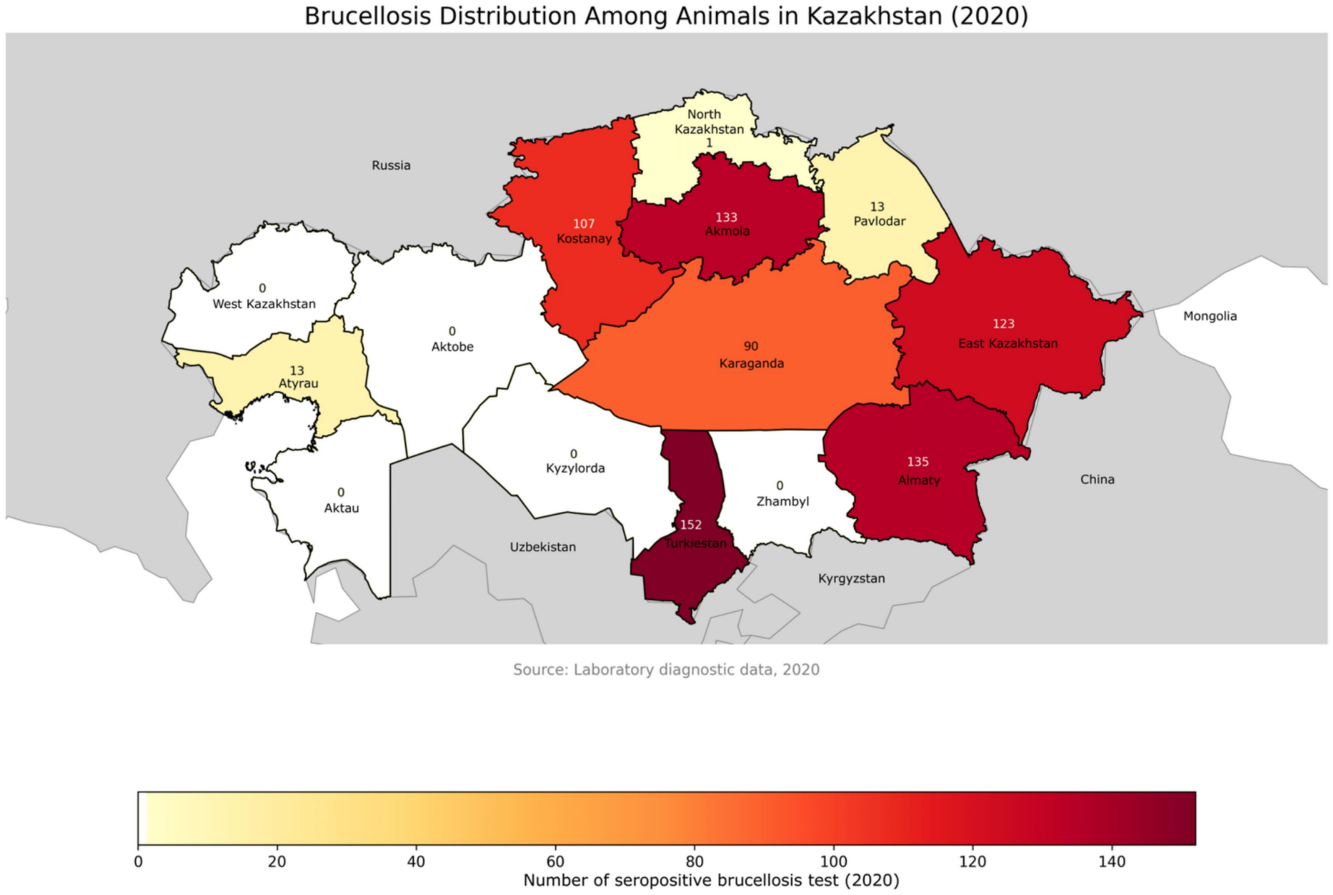
Geographical distribution of brucellosis-positive animals across administrative regions of Kazakhstan in 2020. The map highlights regional differences in disease detection, with darker shades indicating a higher number of positive cases. Data were obtained from laboratory diagnostic surveillance. Labels represent the number of positive animals per region.

In 2020, serological screening was conducted on 44,835 samples collected from 22,586 animals, yielding 1,863 positive results (4.2%) through various diagnostic methods, which corresponded to 767 seropositive animals Figure 3 (116, 117). The samples were collected using a random sampling approach in accordance with standard veterinary epidemiological guidelines.

**Figure 4:**
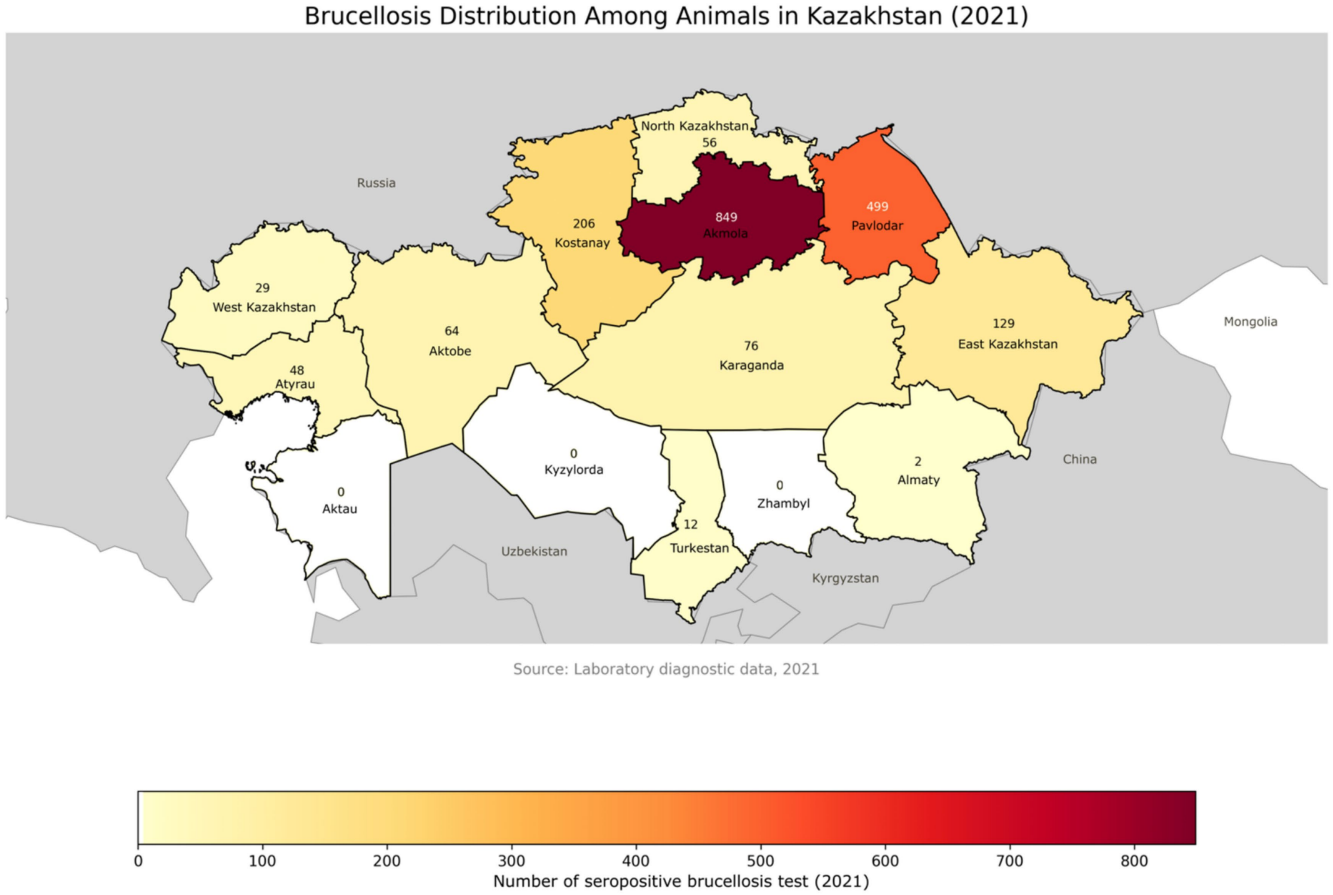
Geographical distribution of brucellosis-positive animals across administrative regions of Kazakhstan in 2021. Compared to 2020, a noticeable increase in the number of positive cases is observed in several regions. The color gradient represents the intensity of detected cases, with regional labels showing the exact number of infected animals.

In contrast, in 2021, although only 28,699 randomly collected samples from 10,008 animals were tested, the proportion of seropositive results increased significantly to 14.9% (4,281 positive results across 1,970 animals) Figure 4 (116, 117).

This notable rise may indicate more targeted sampling within high-risk populations or reflect a genuine increase in the disease burden.

Among serological testing methods, the Rose Bengal Plate Test (RBPT) and enzyme-linked immunosorbent assay (ELISA) consistently demonstrated higher detection rates—20.7 and 20.4%, respectively—compared to the complement fixation test (CFT, 19.1%) and radial immunodiffusion (RID, 6.5%). These findings reinforce previous conclusions that ELISA and RBPT are particularly effective for large-scale screening in endemic areas.

Molecular testing, while more limited in sample volume, demonstrated a substantial increase in detection rates between 2020 (1.2%) and 2021 (12.4%), reinforcing the utility of PCR in confirming active infections, especially when serological results are ambiguous or in early-stage disease. Bacteriological confirmation, considered the gold standard despite its relatively low sensitivity, yielded positive isolates in 2.1% of tested samples in 2020 and 5.0% in 2021, underscoring the importance of culture-based confirmation in outbreak investigations and pathogen genotyping.

Regional analysis during the same period revealed sustained epizootic activity, particularly in Akmola, East Kazakhstan, Almaty, Kostanay, and Karaganda regions, where seroprevalence rates in some zones reached up to 14%. By 2021, new hotspots emerged, including Pavlodar (28.1%) and East Kazakhstan (86.6%), as well as southern regions such as Turkestan (91.7%) and Aktobe (94.9%) (116, 117).

This trend is further supported by national outbreak statistics for 2023, Figure 5. In 2023, a total of 532 brucellosis outbreaks were registered among livestock, exceeding the number reported in 2022 (503 outbreaks) (116, 117).

**Figure 5:**
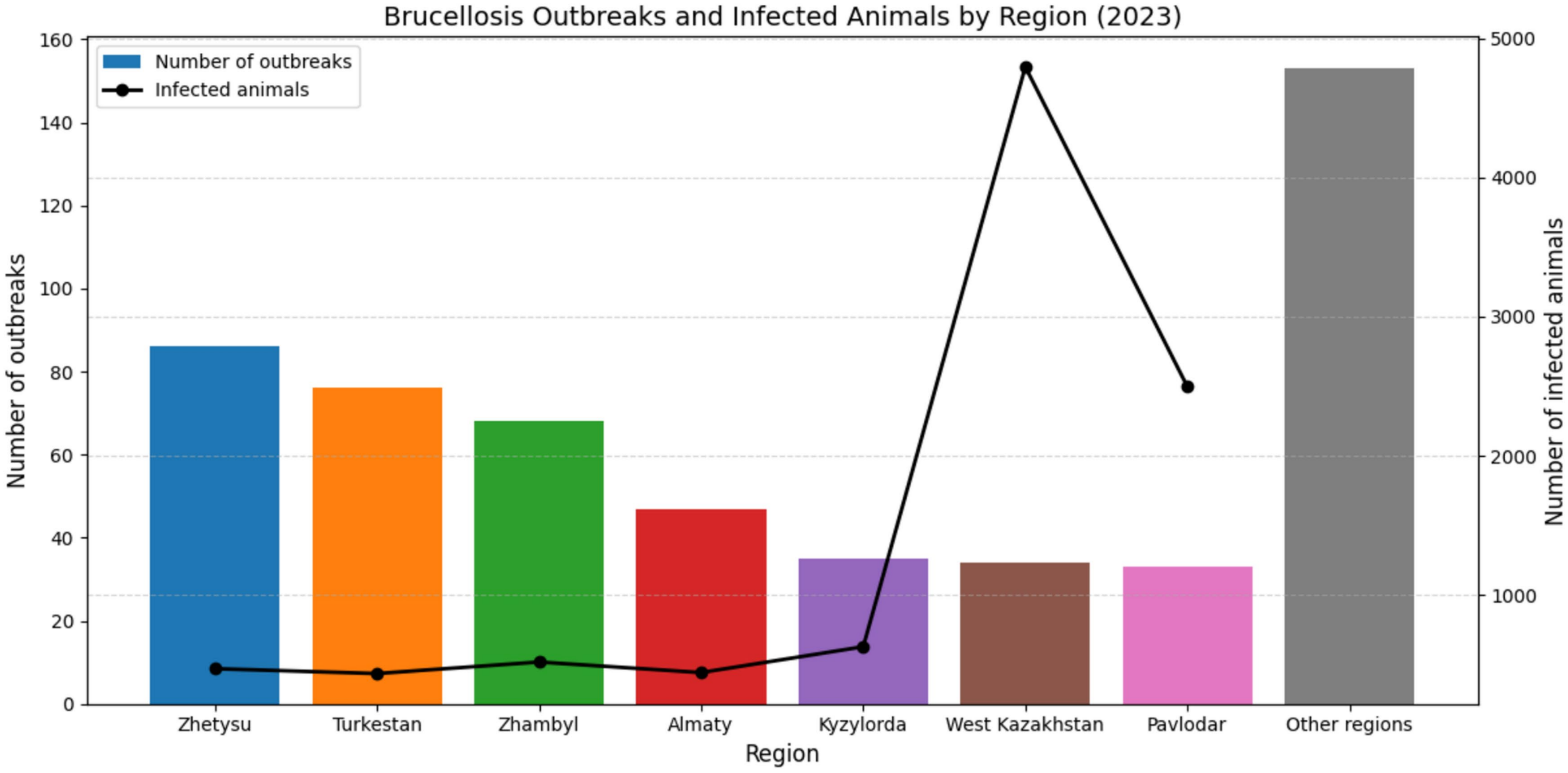
Brucellosis outbreaks and infected animals by region in Kazakhstan 2023.

The highest numbers of confirmed outbreaks were recorded in the Zhetysu region (86 outbreaks, 469 infected animals), followed by Turkestan (76 outbreaks, 434 animals), Zhambyl (68 outbreaks, 517 animals), Almaty (47 outbreaks, 441 animals), Kyzylorda (35 outbreaks, 627 animals), West Kazakhstan (34 outbreaks, 4,796 animals), and Pavlodar (33 outbreaks, 2,496 animals). Notably, 518 of the outbreaks (80.0%) occurred in holdings officially classified as brucellosis-free, indicating a persistent challenge in disease monitoring and control. The majority of infected livestock comprised small ruminants (54%) and cattle (26%), with the remainder being other domestic species or cases with unidentified origin (116, 117). These data underscore the widespread nature of brucellosis in livestock populations and emphasize the continued risk of zoonotic transmission in endemic areas such as southern Kazakhstan, where dense livestock populations and traditional animal husbandry practices persist.

##### 2.2.5.1 Diagnostic methods and control strategies

Brucellosis, a widespread zoonotic disease, affects both humans and animals, particularly in endemic regions (118, 119). Diagnostic methods for brucellosis include culturing, serology, and molecular techniques, with culture remaining the gold standard (118, 120). However, serological tests are commonly used in endemic areas due to their cost-effectiveness and ease of use (118, 121). PCR-based methods offer rapid and sensitive diagnosis (119). In small ruminants, *Brucella melitensis* and *Brucella ovis* are the primary causative agents, with distinct characteristics and diagnostic approaches. The complex nature of brucellosis, including cross-reactivity with other bacteria, necessitates a thorough understanding of Brucella antigens and host immune responses for effective diagnosis and control (121, 122).

Genetic resistance to brucellosis in sheep is an emerging focus in breeding programs aimed at improving disease resilience. In a study by Li et al. (123), whole-genome resequencing of F₂ offspring from Dorper × Hu sheep identified 205 single nucleotide polymorphisms (SNPs) associated with resistance to brucellosis. Candidate genes were significantly enriched in pathways related to cell adhesion and immune regulation, including the Hippo signaling pathway and cell adhesion molecules. These findings provide valuable insights into the genetic mechanisms underlying brucellosis resistance and offer potential molecular markers for selective breeding strategies (123).

##### 2.2.5.2 Vaccination and epidemiological monitoring

Vaccination is a key control strategy, with the Rev.1 vaccine showing 80% effectiveness in preventing abortions in pregnant ewes (124). However, vaccination coverage and timing are critical factors influencing human brucellosis rates (125). A large-scale study in Kuwait revealed high seroprevalence in unvaccinated herds, emphasizing the need for immediate control measures (126). While Rev.1 is the most suitable vaccine, its use is limited due to false-positive reactions in serological tests. Alternative vaccines, such as the *B. melitensis* B115 strain, have been explored but showed significant abortifacient effects in pregnant ewes (127).

Thus, effective epidemiological monitoring requires a comprehensive approach that combines regular testing, seroprevalence analysis, evaluation of genetic and management factors, as well as coordination of actions at the national level through specialized reference laboratories. These measures allow for the well-founded development of biosecurity strategies, prevent the spread of diseases, and promote the improvement of livestock health.

### 2.3 Hereditary diseases

Hereditary diseases in sheep have been documented since at least the 18th century, with scrapie being one of the earliest recognized conditions with a genetic component. First documented in Britain in 1772 and possibly present since 1720, scrapie research suggests the prime cause is hereditary, affecting only individuals of the homozygous recessive type (ss as opposed to sS and SS), though affected animals also harbor a transmissible scrapie agent (128).

Genetic disorders in sheep range from monogenic to multifactorial diseases. As noted by Jolly et al. (129), most inherited disorders in sheep are autosomal recessive traits, as dominantly inherited disorders tend not to be propagated due to their expression in breeding animals. The incidence of particular disorders may be higher than in human populations due to greater relatedness between mated animals and the hierarchical nature of livestock breeding.

Liver metabolism disorders documented in sheep include hyperbilirubinaemia with photosensitivity in Southdown sheep, inherited as an autosomal recessive trait (129), and a similar disease in Corriedale sheep with different pathophysiology (130). Both demonstrate environmental factors (chlorophyll-containing diet and sunlight) working with genetic defects to cause disease.

Several lysosomal storage diseases have been identified in sheep, including glycogen storage disease Type II in Corriedales (131), GM1 gangliosidosis (132), and ceroid-lipofuscinosis in South Hampshire sheep (extensively studied as a model for human disease). These disorders are characterized by accumulation of undigested substrates within lysosomes and are typically inherited as autosomal recessive traits.

Neurological disorders in sheep include lower motor neuron disease in Romney sheep (133), neuroaxonal dystrophy in multiple breeds, ovine spongiform leucoencephalopathy in New Zealand Romneys (134), cerebellar cortical atrophy in Corriedale and Drysdale lambs, and the “star-gazing” syndrome in Coopworth lambs that resembles a similar disorder in Border Leicester sheep.

Integumentary system disorders include Ehlers-Danlos syndrome causing skin fragility, epidermolysis bullosa in Suffolk and South Dorset Down flocks (135), and wool abnormalities such as the dominantly-inherited lustrous wool gene in Romney sheep. Osteogenesis imperfecta with skin fragility has been documented in New Zealand Romney flocks (136).

The study of hereditary diseases in sheep not only contributes to improved sheep health and production but also provides valuable insights into analogous human genetic disorders through comparative medical research. A search of available scientific literature revealed no published studies on the genetic basis of hereditary diseases in sheep in Kazakhstan. As outlined in Table 3, various hereditary diseases, such as spider lamb syndrome, are linked to specific genetic mutations with varying inheritance patterns. Understanding these diseases can offer a deeper understanding of genetic disorders in both veterinary and human medicine.

**Table 3:** Hereditary diseases in sheep: genetic mutations and inheritance types.

Disease / syndrome	Genetic cause/Mutation	Main symptoms	Breeds affected	Inheritance
Spider lamb Syndrome	Mutation in *FGFR3* gene (codon 700)	Skeletal deformities, long limbs, lethality	Suffolk, Hampshire	Autosomal recessive
Entropion	Polygenic, some evidence on chromosome 15	Inward-turning eyelids, eye irritation	Swiss White Alpine, others	Polygenic
Cryptorchidism	Likely polygenic	Undescended testicle(s), infertility	Various	Polygenic
Congenital muscular dystrophy	Mutation in *LAMA2* gene	Muscle weakness, stunted growth	Texel (rare)	Autosomal recessive
Dermatosparaxis (Ehlers-Danlos type)	Mutation in *ADAMTS2* gene	Fragile, easily torn skin	White Dorper, Norwegian White	Autosomal recessive
Heritable cataracts	Likely associated with ovine chromosome 6	Clouded lens, impaired vision	Romney, Merino	Likely recessive or dominant
β-Mannosidosis	Deficiency of β-mannosidase enzyme	Neurological signs, developmental delay	Nubian goats, some sheep	Autosomal recessive

#### 2.3.1 Spider lamb syndrome

Spider Lamb Syndrome (SLS), or ovine hereditary chondrodysplasia, is a congenital, semi-lethal disorder of the musculoskeletal system characterized by severe skeletal malformations. Typical clinical manifestations include elongated limbs, spinal curvature (kyphoscoliosis), deformities of the ribs and sternum, Roman nose, marked muscular atrophy, and absence of subcutaneous fat. Radiographic and histological examinations reveal impaired endochondral ossification, with enlarged zones of proliferating and hypertrophic chondrocytes and disorganized cellular architecture within the growth plates of long bones and vertebrae (137).

Genetically, the syndrome is inherited in an autosomal recessive manner and has been mapped to the distal region of ovine chromosome 6 (OAR6). The principal candidate gene implicated in the pathogenesis of SLS is fibroblast growth factor receptor 3 (*FGFR3*)—a transmembrane tyrosine kinase receptor that plays a key role in regulating bone growth by limiting chondrocyte proliferation within the growth plate. Loss-of-function mutations in *FGFR3*, as demonstrated in animal models such as mice, result in excessive bone growth, expansion of hypertrophic zones, and disruption of the orderly arrangement of chondrocytes—findings that closely parallel the histopathological features observed in SLS-affected lambs (138). Thus, Spider Lamb Syndrome serves as a valuable model for studying skeletal dysplasias associated with *FGFR3* dysfunction and contributes to a broader understanding of bone development and inherited osteochondrodysplasias in mammals.

#### 2.3.2 Entropion

Entropion, an inward rolling of the eyelid, is a congenital disorder in sheep with a genetic component (19, 139). It affects various breeds, with some showing higher prevalence and severity (140). Early diagnosis and treatment are crucial to prevent complications. Treatment options include manual eversion with antibiotic ointment for mild cases and Michel wound clamps for more severe cases. Selective breeding may help reduce the incidence of entropion in sheep populations (141). Genome-wide association studies have identified several genetic regions associated with entropion in sheep. A study on Columbia, Polypay, and Rambouillet breeds found significant associations on chromosomes 6, 1, 2, 13, and 16 (19). Further research revealed genome-wide significant SNPs in *SLC2A9*, along with suggestive associations in *PIK3CB*, *KCNB1*, *ZC3H12C*, *JPH1*, and *MYO3B* (19). In Swiss White Alpine sheep, a significant association was found on chromosome 15, with *SMTNL1* and *CTNND1* as potential candidate genes (139). However, subsequent analysis suggested that entropion may be a complex disease caused by non-coding regulatory variants rather than protein-changing mutations in these genes (139). These studies represent the first genome-wide analyses of entropion-associated gene regions in any mammalian species, providing valuable insights for future research and potential marker-assisted selection in sheep breeding.

#### 2.3.3 Cryptorchidism

Cryptorchidism, the failure of one or both testes to descend into the scrotum, is a common condition in sheep. Studies have shown varying incidence rates, from 0.56% in young male lambs (142) to 7.4% in North Ronaldsay sheep (143). Unilateral cryptorchidism is more common than bilateral, with the right testis more frequently affected. Abdominal retention is more common than inguinal in sheep (142). The process of testicular descent involves three phases: abdominal translocation, transinguinal migration, and inguinoscrotal migration, with various hormones and structures playing crucial roles (144). Laparoscope-assisted orchiectomy has been successfully used to treat cryptorchidism in sheep (145).

Using integrated transcriptomic and proteomic approaches, recent study by Sheng-Wei Pei et al. (13) revealed substantial changes in gene and protein expression profiles between undescended (cryptorchid) and normal testes. Key downregulated genes were associated with spermatogenesis and male gamete development, while upregulated genes were linked to apoptotic and metabolic processes. Importantly, several candidate genes—including *AKAP4*, *AKAP3*, *FSIP2*, and *HSPA1A*—were identified as potentially involved in the disruption of testicular descent and function. These findings provide novel insights into the molecular pathways and genetic factors contributing to unilateral cryptorchidism in sheep and offer a basis for further genetic screening and breeding strategies (13).

#### 2.3.4 Congenital muscular dystrophy

Ovine congenital progressive muscular dystrophy (OCPMD) is a genetic disorder affecting Merino sheep, characterized by hind limb stiffness and progressive gait abnormalities (14, 146). Initially described in the 1960s and 1970s, OCPMD primarily affects the extensors of the hip, stifle, and hock joints, as well as flexors of the digits (14). Histopathological examination revealed dystrophic changes in type I myofibers and the presence of nemaline bodies (146). Recent advances in the genetics of congenital myopathies have highlighted the role of specific structural protein defects in disease pathogenesis. A single base deletion in the *TNNT1* gene, which encodes slow skeletal troponin T, has been identified as the causative mutation in ovine congenital progressive muscular dystrophy (OCPMD), effectively reclassifying this disorder as a form of *TNNT1*-related congenital myopathy (146). Similarly, laminin α2 Chain-Deficiency, resulting from mutations in the gene encoding the laminin α2 subunit (*LAMA2*), causes congenital muscular dystrophy characterized by progressive muscle degeneration and peripheral neuropathy. Therapeutic delivery of linker mini-genes such as *αLNNd* or its truncated form *αLNNdΔG2′* has shown functional and histological improvements in *dy2J/dy2J* mouse models by restoring laminin polymerization, enhancing grip strength, and promoting myelination (via AAV-mediated expression) beyond muscle tissue into peripheral nerves (147). Together, these findings underscore the critical role of sarcolemmal and extracellular matrix proteins in maintaining muscle integrity and highlight the promise of targeted gene therapy approaches for diverse forms of hereditary muscular disorders.

#### 2.3.5 Dermatosparaxis (Ehlers-Danlos type)

Dermatosparaxis is a rare autosomal recessive connective tissue disorder reported in White Dorper sheep populations in Brazil (148). Clinically, the condition manifests as pronounced skin fragility and hyperextensibility, attributed to dysplastic collagen fiber development (148, 149). Histopathological evaluation typically reveals disorganized and fragmented collagen bundles, along with dermal hemorrhagic foci (16, 149). The molecular basis of the disorder has been identified as a missense mutation (c.421G > T) in the *ADAMTS2* gene, which disrupts the function of the enzyme’s catalytic domain (16). This mutation is detectable via PCR and DNA sequencing, enabling early molecular diagnosis and the implementation of informed breeding strategies to prevent further transmission (16). Notably, dermatosparaxis in sheep exhibits strong parallels to Ehlers-Danlos syndrome type VIIC in humans, emphasizing its significance as a model in comparative and translational medicine (149).

#### 2.3.6 Heritable cataracts

An autosomal dominant form of inherited cataract has been characterized in New Zealand Romney sheep, serving as a valuable large animal model for the study of human cataractogenesis (150). The disease is characterized by progressive lens opacity resulting from degenerative changes in lens fiber architecture and composition. The condition typically manifests postnatally, with focal cortical opacities appearing at 1–2 months of age and progressing to complete lens opacity by approximately 10–11 months (150). Genetic mapping studies have localized the cataract-associated locus to ovine chromosome 6, with *AFF1* proposed as a candidate gene (151). Histological and ultrastructural analyses of affected lenses reveal progressive degeneration of lens fibers, cytoplasmic vacuolation, and alterations in lens ion homeostasis during disease progression (150). Owing to its well-characterized clinical progression and genetic basis, this ovine model serves as a valuable tool for translational research in cataract therapy. Notably, it has been effectively employed in the preclinical evaluation of therapeutic agents. For instance, the macrocyclic calpain inhibitor CAT811 demonstrated efficacy in delaying cataract formation when administered topically, underscoring the model’s significance in the development of potential pharmacological interventions (152).

#### 2.3.7 β-Mannosidosis

β-Mannosidosis is a hereditary disorder of glycoprotein catabolism, caused by β-mannosidase deficiency and the accumulation of specific oligosaccharides (153). The condition has been extensively studied in goats, where affected neonates present with a distinctive phenotype, including inability to stand, intention tremor, nystagmus-like eye movements, deafness, and various skeletal anomalies (153, 154). Pathological findings include widespread cytoplasmic vacuolation, reduced myelination, and ventricular dilatation (153). Sequencing of the caprine β-mannosidase cDNA revealed high homology with the bovine counterpart (155). A single-base deletion in the coding sequence was identified as the causative mutation in affected goats, leading to a truncated protein. This discovery has facilitated the development of genetic tests for carrier detection and prenatal diagnosis, as well as the assessment of stem cell engraftment as a potential therapeutic strategy (155).

## 3 Recommendations and future perspectives

The complex landscape of sheep diseases in Kazakhstan necessitates a multifaceted approach to disease management that leverages both conventional veterinary practices and cutting-edge genetic technologies. Based on our comprehensive review of parasitic, infectious, and hereditary diseases affecting Kazakhstani sheep populations, we propose the following strategic recommendations for enhancing disease resistance and improving flock health.

### 3.1 Integration of molecular genetic technologies in breeding programs

The implementation of advanced molecular genetic tools represents a promising avenue for enhancing disease resistance in Kazakhstani sheep breeds. Genome-wide association studies (GWAS), quantitative trait loci (QTL) mapping, and SNP microarray analyses have demonstrated considerable potential for identifying genetic markers associated with resistance to various pathogens. The findings by Li et al. (17) establishing correlations between MHC gene polymorphisms and echinococcosis resistance in Chinese Merino sheep provide a methodological framework that could be adapted to study indigenous Kazakhstani breeds (17).

We recommend the establishment of a national genomic database for Kazakhstani sheep breeds, incorporating comprehensive phenotypic and genotypic data with particular emphasis on disease-related traits. This database would facilitate large-scale GWAS and QTL studies aimed at identifying breed-specific resistance alleles and haplotypes, particularly those associated with economically significant conditions such as brucellosis, foot rot, and parasitic infections endemic to the region.

### 3.2 Development of marker-assisted selection protocols

The European experience in combating transmissible spongiform encephalopathies (TSE), including scrapie, offers valuable insights for Kazakhstan. The Ram Genotyping Scheme implemented in the United Kingdom demonstrated that selective breeding based on *PRNP* genotypes could substantially reduce scrapie prevalence. Adapting similar approaches to the Kazakhstani context would involve developing standardized protocols for genotyping breeding stock and establishing selection criteria that prioritize resistant variants while maintaining desirable production traits.

Recent research by Cinar et al. (56) identifying *GBP6* and *TCHH* genes as pivotal determinants of foot rot resistance underscores the potential of molecular markers for enhancing disease resilience. We recommend initiating pilot marker-assisted selection programs focusing initially on diseases with well-characterized genetic resistance mechanisms, such as scrapie and parasitic infections, with subsequent expansion to cover a broader spectrum of pathogens as additional genetic associations are identified.

### 3.3 Epidemiological modeling and selection impact assessment

The implementation of genetic selection for disease resistance necessitates a thorough understanding of its epidemiological implications. Bishop and Stear (156) “closed cycle” concept suggests that reducing infection pressure through selective breeding creates a positive feedback loop by decreasing immune challenges, which subsequently diminishes pathogen transmission rates. Experimental data indicate that such epidemiological changes may exceed predictions based solely on classical genetic theory.

We recommend developing Kazakhstan-specific epidemiological models that integrate genetic selection parameters with environmental variables, husbandry practices, and pathogen characteristics. These models would enable more accurate prediction of selection outcomes and facilitate the optimization of breeding strategies to maximize population-level disease resistance while minimizing potential adverse effects on productivity or susceptibility to other pathogens.

### 3.4 Capacity building and knowledge transfer

The successful implementation of genetic resistance breeding programs in Kazakhstan requires substantial investment in human capital development. We recommend establishing specialized training programs for veterinarians, animal scientists, and farmers focusing on:

- Molecular genetic principles underlying disease resistance;
- Sample collection and preservation protocols for genetic analysis;
- Interpretation and application of genomic data in breeding decisions;
- Integration of genetic selection with conventional disease control measures.

Collaboration with countries that have successfully implemented similar programs, such as Australia, New Zealand, and the United Kingdom, could accelerate knowledge transfer through exchange programs, joint research initiatives, and technical workshops.

### 3.5 Regulatory framework and infrastructure development

The establishment of a supportive regulatory environment is essential for the sustainable implementation of genetic resistance breeding programs. We recommend developing national standards for genetic testing, certification protocols for resistant breeding stock, and incentive mechanisms to encourage farmer participation in resistance breeding initiatives.

Furthermore, investment in laboratory infrastructure capable of high-throughput genotyping and phenotyping is crucial for scaling up genetic selection efforts. The establishment of regional diagnostic centers equipped with advanced molecular technologies would facilitate routine screening of breeding animals and contribute to comprehensive disease surveillance throughout Kazakhstan.

### 3.6 Holistic approach to disease management

While genetic selection offers considerable promise for enhancing disease resistance, it should complement rather than replace conventional control measures. We recommend developing integrated disease management protocols that combine genetic selection with strategic vaccination, targeted anthelmintic treatments, improved biosecurity practices, and optimized nutrition to maximize flock health and productivity.

The unique environmental conditions of Kazakhstan, characterized by extreme continental climate and extensive grazing systems, necessitate context-specific disease management strategies that account for regional variations in pathogen prevalence, breed characteristics, and husbandry practices. Pilot implementation of integrated approaches in representative regions would generate valuable data for subsequent nationwide deployment.

## 4 Conclusion

The intersection of veterinary epidemiology, molecular genetics, and animal breeding presents unprecedented opportunities for enhancing disease resistance in Kazakhstani sheep populations. Our comprehensive review of parasitic, infectious, and hereditary diseases affecting these animals reveals both significant challenges and promising avenues for intervention.

Kazakh sheep breeds have evolved under extreme continental climatic conditions, characterized by sharp temperature fluctuations, limited feed resources, and the historical nomadic lifestyle of their owners. This evolutionary history has subjected these populations to strong natural selection pressures, favoring individuals with enhanced resilience to environmental stressors, pathogens, and resource limitations. Such resilience may also reflect a degree of ecological plasticity in key genetic markers, allowing these breeds to maintain adaptive responses across diverse environmental contexts.

The prevalence of economically significant conditions such as echinococcosis, fascioliasis, brucellosis, and foot rot underscores the urgent need for effective disease management strategies. While conventional approaches relying on chemical treatments and vaccination programs have yielded limited success, the integration of genetic selection for enhanced resistance offers a sustainable complementary approach that could substantially reduce disease burden while minimizing reliance on therapeutic interventions.

The identification of genetic markers associated with resistance to various pathogens, particularly polymorphisms in the MHC complex and *PRNP* gene, provides a foundation for developing marker-assisted selection programs tailored to Kazakhstani breeds. However, the absence of comprehensive studies on the genetic basis of disease resistance in local sheep breeds remains a critical bottleneck. This deficiency not only limits the development of targeted breeding strategies but also puts Kazakhstan at a disadvantage compared to countries actively integrating genomic tools into animal health programs.

The successful implementation of disease resistance breeding programs demands close collaboration between research institutions, veterinary services, and farming communities. International experience demonstrates that such collaborative approaches, combining scientific expertise with practical knowledge, yield the most significant and sustainable improvements in flock health and productivity.

In conclusion, research on genetic disease resistance offers significant prospects for enhancing the productivity and sustainability of Kazakhstan’s sheep industry. The adaptation of international methodologies, integration of cutting-edge genetic technologies, and development of breeding programs tailored to the unique characteristics of local breeds will enable reductions in disease impact, enhancement of export potential, and advancement of the industry’s sustainable development. By addressing the complex interplay between host genetics, pathogen dynamics, and environmental factors, Kazakhstan can establish resilient sheep production systems capable of meeting both current demands and future challenges in an ever-changing global landscape.
